# Case Report: Malignant perivascular epithelioid cell tumor with aggressive mediastinal invasion and pulmonary metastasis

**DOI:** 10.3389/fonc.2025.1551663

**Published:** 2025-08-18

**Authors:** Daniel F. Leach, Srivikram Margam S, Marissa Foster, Jarrod B. Adkison

**Affiliations:** ^1^ Department of Radiation Oncology, The Ohio State University, Columbus, OH, United States; ^2^ Family Medicine, Ventura County Medical Center, Ventura, CA, United States; ^3^ Department of Radiation Oncology, Indiana University School of Medicine, Indianapolis, IN, United States; ^4^ Radiation Oncology, Southeast Health, Dothan, AL, United States

**Keywords:** perivascular epithelioid cell tumors, intensity-modulated radiotherapy, 3D conformal radiotherapy, palliative radiotherapy, mTOR inhibitors, VEGF inhibitors

## Abstract

Perivascular epithelioid cell tumors (PEComas) are rare, typically benign soft tissue tumors that can develop at various anatomic sites. Malignant PEComas are rarer entities but may present aggressively with metastasis to the lungs or local recurrence years after initial presentation. In unresectable or metastatic cases, treatment options are limited due to the resistance of PEComas to chemotherapy and radiotherapy. The present case describes a 59-year-old man with a highly aggressive malignant PEComa, which ultimately invaded the mediastinum and replaced the right middle and lower lobes of the lung despite systemic therapy with oral sirolimus and definitive radiotherapy. As only three prior cases have described malignant PEComas invading the mediastinum, we highlight the clinical course of such an aggressive cancer and review current treatment paradigms.

## Introduction

Perivascular epithelioid cell tumors represent a group of rare, typically benign soft tissue tumors of mesenchymal origin characterized by a particular constellation of features on histopathology: epithelioid to spindle morphology, clear to granular cytoplasm, mild nuclear atypia with prominent nucleoli, co-expression of melanocytic markers (i.e., HMB-45, melan-A) and smooth muscle markers (i.e., smooth muscle actin, desmin), and perivascular distribution often with vascular smooth muscle infiltration (1–5). Recent reviews and a retrospective cohort study have identified a preponderance of perivascular epithelioid cell tumor (PEComa) cases among female patients with a relatively low median age in the forties (6, 7). Primary tumor sites vary by source but appear to be the kidney (8, 9), gastrointestinal tract (10), retroperitoneum (11, 12), uterus (13), and pelvic soft tissues (14). However, PEComas can arise from any organ, corroborated by the prevalence of case reports describing various primary tumor locations (4, 15) including the head and neck (16), lungs (17–19), pancreas (20, 21), liver (22, 23), genitourinary tract (24), adrenal glands (25), reproductive tract (26), skin (27), and bone (28).

The PEComa group is comprised of angiomyolipomas, pulmonary/extrapulmonary clear cell tumors, lymphangioleiomyomatosis, clear cell myomelanocytic tumor of the falciform ligament or ligamentum teres, soft tissue clear cell myomelanocytic tumors, abdominopelvic perivascular cell epithelioid sarcomas, renal capsular leiomyomas per some authors, and other tumors with similar characteristics at various anatomic sites broadly termed PEComa-not otherwise specified (PEComa-NOS) per the 2020 WHO classification of soft tissue tumors (1, 29, 30). PEComas have an association with tuberous sclerosis (TS), displaying similar genetic mutations and alterations (1–3) with clinical linkage described by several case reports (31, 32). However, in most cases, PEComas are diagnosed incidentally by diagnostic imaging.

Historically, PEComas have been classified as benign, uncertain malignant potential, malignant potential, and malignant, a scheme initially described by Folpe et al. Non-gynecologic histopathologic criteria include tumor size ≥5 cm, infiltrative pattern, high nuclear grade and cellularity, high mitotic rate (≥1/50 high-power field), necrosis, and lymphovascular invasion with ≥2 features required for malignant classification (33). However, Bleeker et al. showed in a retrospective review of 234 cases that tumor size >5 cm and high mitotic rate were the only pathologic features significantly associated with recurrence after surgical resection, proposing a modified risk classification scheme that removed malignant potential (34). To date, the 2020 WHO classification of soft tissue tumors divides PEComas into benign and malignant types and employs the above non-gynecologic-specific criteria as well as modified gynecologic-specific criteria whereby ≥3 histopathologic features are required for malignant classification (30).

Malignant PEComas can present aggressively with rapid growth, metastasis, and death rates similar to high-grade sarcomas (3, 35). Metastatic patterns commonly occur in the lungs (36, 37), followed by the liver, peritoneum, and bone (12, 38). Additionally, a few cases have reported synchronous presentations with malignant cancers (9, 10, 13, 19). Malignant PEComas with aggressive pulmonary invasion or metastasis are very rare, with a few cases described (39, 40), and may precipitate acute complications such as pneumothorax (41). Overall management of such locally advanced, unresectable, or multiply metastatic cases is unclear, as surgical resection with negative margins has been established as the cornerstone of treatment for malignant PEComas (6).

We report a unique presentation of an unresectable malignant PEComa in a 59-year-old man involving the neck and chest wall who developed mediastinal invasion and replacement of the right middle/lower lobes of the lung despite receiving systemic therapy with sirolimus and definitive hypofractionated radiotherapy (RT). Salvage re-irradiation and pazopanib were planned due to disease progression but could not be carried out due to subsequent acute hypoxic respiratory failure requiring intubation. To our knowledge, only three reports have described the clinical course of malignant PEComas invading the mediastinum (42–44). The present case highlights how aggressive malignant PEComas can behave and the limitations of non-invasive treatment.

## Case presentation

A 59-year-old man presented to an outpatient clinic with a progressively enlarged posterior neck/upper back mass. He reported right upper extremity radiculopathy and a 30-lb weight loss over a period of 6 months. Physical exam revealed a soft, non-tender, 15 cm × 15 cm mass extending from the right clavicle up 8 cm along the lateral neck to the superior aspect of the right scapula and medial upper thoracic spine. Cranial nerves were intact. Upper extremity strength was equal and symmetric with a full range of motion bilaterally.

Comprehensive metabolic panel was unremarkable. Complete blood count showed microcytic anemia with a hemoglobin level of 10 g/dL and a mean corpuscular volume of 71 fL without clinical blood loss. Platelets were elevated at 563,000/µL favoring iron deficiency anemia, but iron studies were more consistent with anemia of chronic disease. Computed tomography (CT) chest showed a 6.2-cm × 5.5-cm right superior mediastinal mass with a partially visualized supraclavicular mass. CT neck showed a 14.3-cm × 13.3-cm heterogeneously enhancing mass at the base of the right neck extending along the posterior chest wall. Ultrasound-guided biopsy of the periscapular mass showed epithelioid cells in nests and sheets surrounding fragile blood vessels with accompanying necrosis. Immunohistochemistry (IHC) was positive for HMB-45, desmin, cathepsin K, and E-cadherin; TFE3 staining was not reported. Due to tumor size, presence of necrosis, and hallmark IHC findings, the biopsy was interpreted as a multifocal T3N0M0G3, clinical stage IIIB, malignant PEComa by the American Joint Committee on Cancer’s staging system. Positron emission tomography (PET)/CT showed a 12.8-cm mass extending inferiorly from the right posterior lower neck to the right scapula and a 6.4-cm right anterior mediastinal mass extending into the right upper/middle lobes of the lung. Both masses were intensely ^18^F-2-deoxy-D-glucose (FDG) avid. Of note, there were three pulmonary nodules, raising concern for pulmonary metastasis, especially due to enlargement on subsequent imaging.

As the mass intimately involved structures of the neck and mediastinum, it was deemed unresectable, so the patient was started on sirolimus 4 mg daily. However, the sirolimus level became supratherapeutic at 30.6 ng/mL, so the sirolimus dose was decreased to 2 mg daily.

The patient was briefly hospitalized after his initial diagnosis for acute on chronic anemia requiring transfusion. Hemoccult was positive for which he was started on intravenous pantoprazole. Esophagogastroduodenoscopy and colonoscopy showed no active source of gastrointestinal bleeding. CT chest did not reveal intrathoracic hemorrhage, only showing modest enlargement of the right upper posterior chest wall mass (now 13.2 cm × 7 cm) and the right anterior mediastinal mass (now 6.7 cm × 8.4 cm). Stable left lung nodules were also noted. Consequently, he was discharged on oral pantoprazole with supportive transfusions to maintain a hemoglobin level greater than 8 g/dL. A capsule endoscopy was recommended but ultimately not performed.

Definitive hypofractionated intensity-modulated radiation therapy (IMRT) to the right low posterior neck and right posterior chest wall was initiated with a dose limit of 54 Gy in 30 fractions at the field periphery, due to close proximity to the spinal cord, and a simultaneous integrated boost to 66 Gy in 30 fractions to reach an EQD2 of 70 Gy. The spinal cord received a maximum dose of 39 Gy. The treatment course was complicated by right plexopathy manifesting as right-hand weakness, and two additional admissions for acute on chronic anemia requiring transfusion, although no definite source of blood loss could be identified.

Subsequent CT neck was concerning for disease progression, showing enlargement of the heterogeneous, right posterior cervical neck/chest wall mass (now 16.4 cm × 17.1 cm) with associated osseous erosion of C7-T2 and epidural extension via the right neural foramina with dural sac displacement along C6-T3. Additionally, CT chest, abdomen, and pelvis showed increased size of the right mediastinal/pericardiophrenic mass (now 12.6 cm × 9.1 cm) with a large right pleural effusion and resultant mass effect on the right middle lobe, right atrium, and right mediastinal/hilar vascular structures.

Follow-up magnetic resonance imaging (MRI) of the cervical, thoracic, and lumbar spine redemonstrated the multilobulated, heterogeneous, right lower neck/posterior upper chest mass now with central necrosis extending inferiorly along the right posterior thorax to the level of the T7 vertebral body. Moreover, there was epidural extension through bilateral neural foramina from C6-T4, circumferential tumor involvement causing severe cord compression from T1-T4, and extension of the tumor into the C6-T1 vertebrae and right-sided ribs. The patient was started on dexamethasone to reduce spinal cord edema and compression. Neurosurgical intervention was deemed to entail excessive risk due to the tumor vascularity, aggressive nature, and likelihood of recurrence. Palliative radiotherapy was also considered to relieve cord compression, but the risk of radiation-induced myelopathy was judged to outweigh the potential benefit of tumor size reduction. Thoracic surgical intervention was deemed inappropriate due to the perceived lack of curative or palliative benefit justifying a procedure.

**Figure 1:**
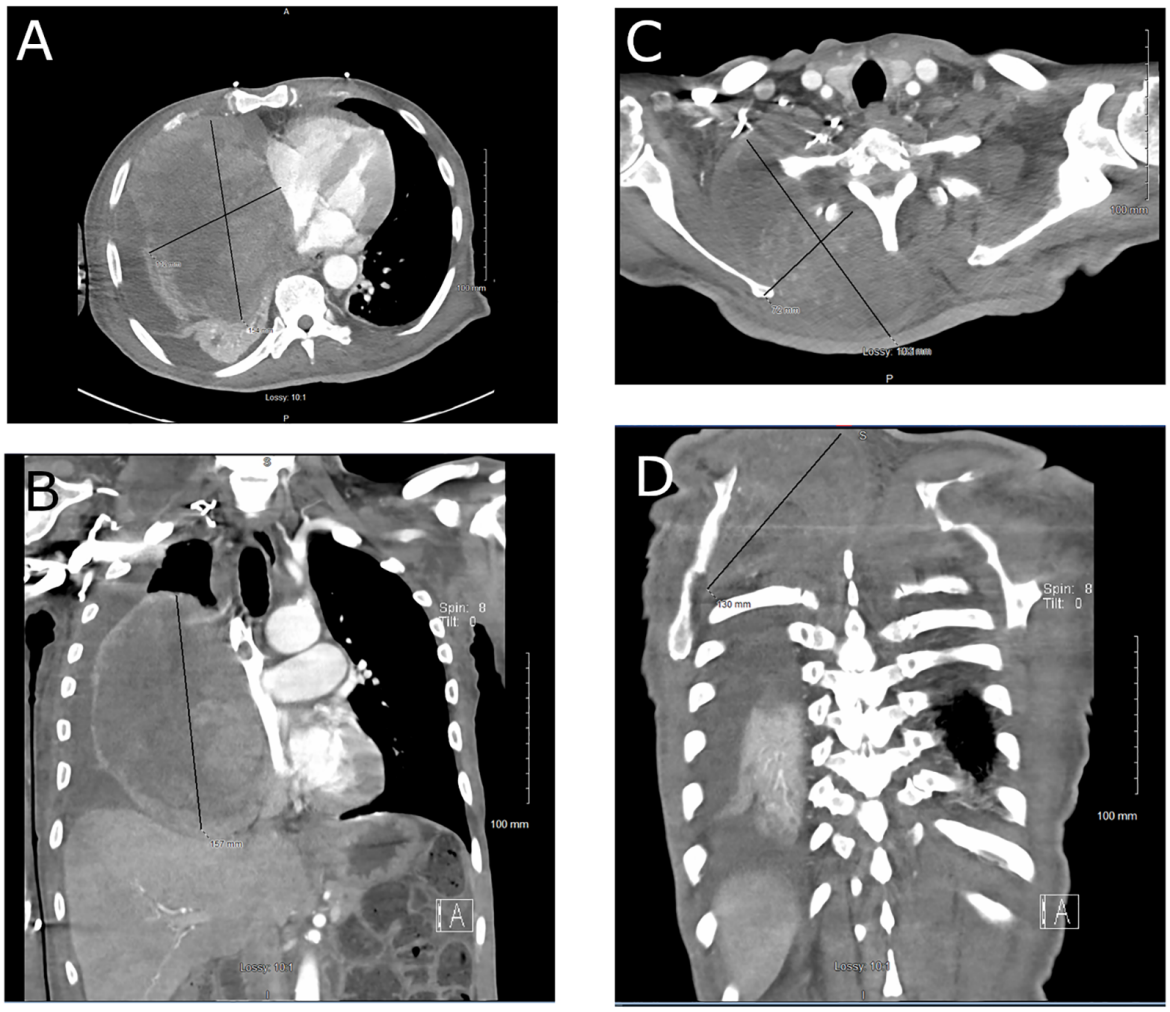
CT chest: **(A)** axial view and **(B)** coronal view, demonstrating replacement of the right middle and lower lobes of the lung by a large malignant PEComa. CT neck: **(C)** axial view and **(D)** coronal view, showing the right dorsal component of the tumor displacing the right scapula.

As such, a 6-week course of pazopanib and re-irradiation with hypofractionated IMRT to 60 Gy in 20 fractions to the chest were planned to relieve the mediastinal compression, but the patient developed acute hypoxic respiratory failure requiring intubation as well as circulatory shock, necessitating continuous vasopressor support. CT chest, abdomen, and pelvis revealed the etiology as secondary to near-total collapse of the right lung with moderate right malignant pleural effusion, collapse of the right heart chambers with leftward mediastinal shift, and numerous pulmonary metastases due to the large, heterogeneous, soft tissue tumor (now 15 cm × 16 cm) in the right intrathoracic chest. The right dorsal back component of the tumor measured 14 cm × 13 cm at that time, causing displacement of the right scapula (**Figure 1**).

**Figure 2:**
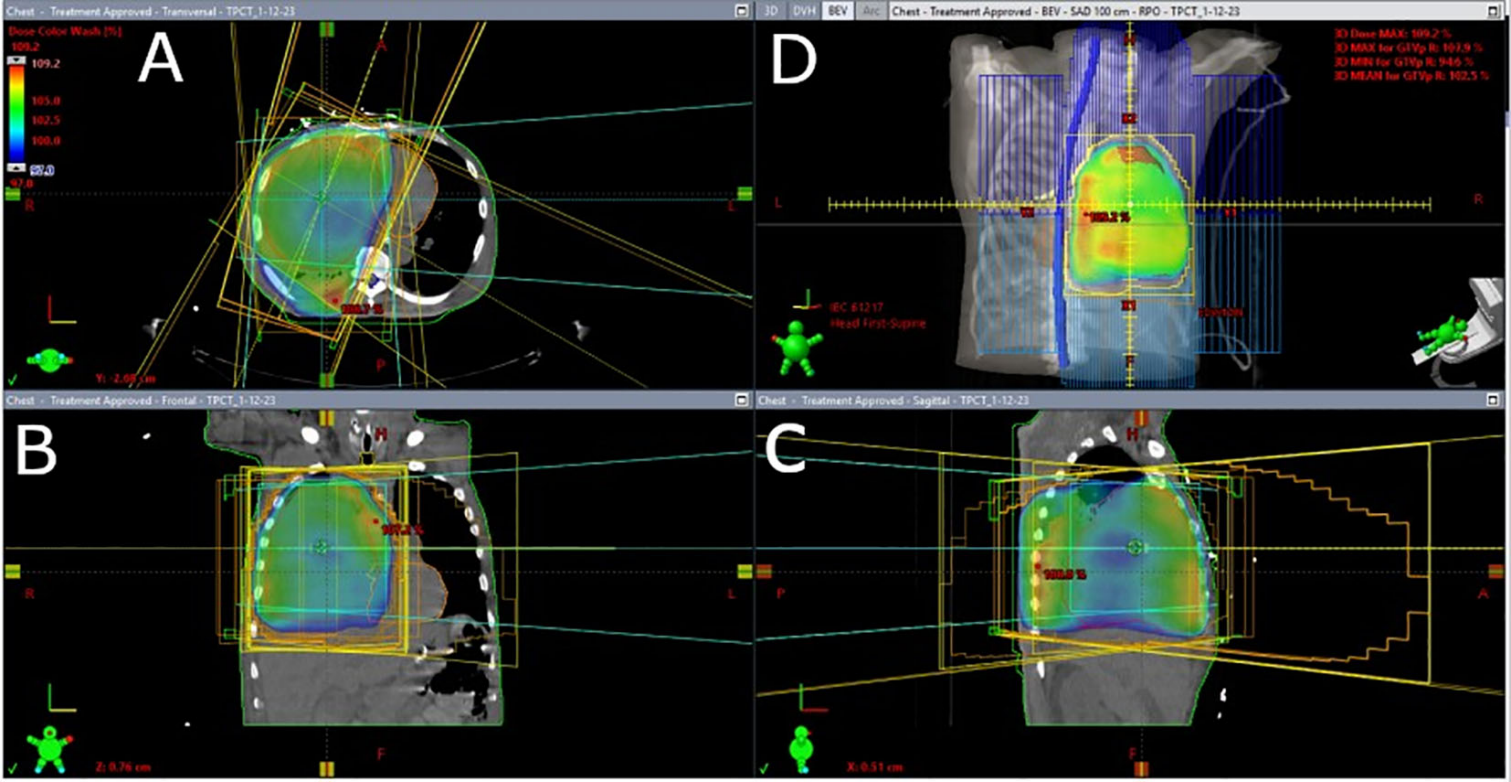
Palliative 3D conformal radiation treatment plan to the right upper lobe, right lower lobe, and right hilum. Color wash depicts the calculated 3D dose distribution: **(A)** axial view, **(B)** coronal view, **(C)** sagittal view, and **(D)** beam’s eye view, showing a treatment volume encompassing the majority of the right lung to treat progressive gross disease. Three prescription dose levels are depicted: blue areas received 100% of the prescription dose, 20 Gy; green areas received 102%–105% of the prescription dose; and red areas received 109% of the prescription dose with a global point maximum (Dmax) of 109.2% landing in an inferior right intercostal space.

**Figure 3:**
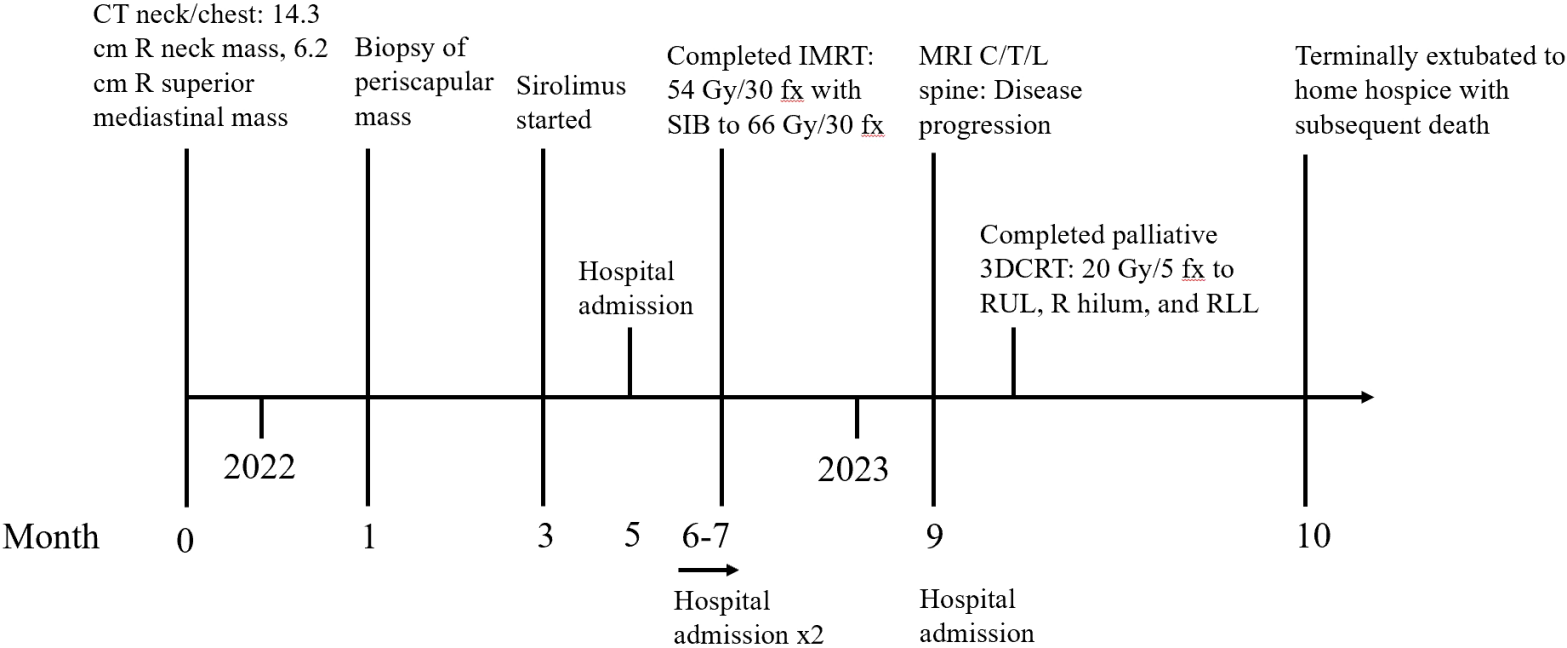
Patient treatment timeline.

With the patient intubated, oral administration of pazopanib was not feasible. As such, palliative 3D conformal radiotherapy to 20 Gy in 5 fractions was initiated to the thoracic component of the tumor to relieve the respiratory symptoms caused by mediastinal invasion (**Figure 2**). Upon extubation, the patient failed a fiberoptic endoscopic evaluation of swallowing and thus was evaluated for percutaneous endoscopic gastrostomy (PEG) tube placement to permit administration of pazopanib. However, review of prior CT findings revealed significant esophageal compression secondary to mediastinal mass effect, so PEG tube placement could not be performed. The patient was reintubated shortly afterward due to worsening hypoxemic and hypercapnic respiratory failure. Given the poor prognosis and inability to proceed with pazopanib therapy, the patient and his family opted for hospice. He was terminally extubated to bilevel positive airway pressure and discharged to home hospice, ultimately passing away (**Figure 3**).

## Discussion

On a molecular level, PEComa pathogenesis has two potential major pathways. The first is linked to mutations in *TSC1* and *TSC2*, genes also involved in the genesis of TS. Despite the lack of cases reporting *TSC* information, existing evidence shows that *TSC* genes were frequently, consistently, and significantly associated with PEComa pathogenesis, likely through an induction of cell proliferation by activation of the mammalian target of rapamycin (mTOR) pathway (45). The second major pathway is the *TFE3* gene translocation; *TFE3* is a gene encoding the broadly expressed transcription factor E3. Aberrant immunoreactivity for TFE3 protein has been previously observed in the vast majority of PEComas; subsequent analysis of the gene status revealed a distinct subset of PEComa cases with *TFE3* gene fusions. Overexpression of *TFE3* mediates expression of cathepsin K, which may represent an IHC marker useful in the identification of *TFE3*-altered PEComas (4). While cathepsin K was positive in our patient’s biopsy specimen, *TFE3* expression or translocation was not reported. Two cases with malignant *TFE3*-rearranged PEComa showed treatment response with the antivascular endothelial growth factor (VEGF) receptor tyrosine kinase inhibitor apatinib, suggesting that VEGF signaling may also be implicated in *TFE3*-associated PEComas (46, 47). With respect to *TFE3* expression, a retrospective review of 29 patients showed that *TFE3* overexpression correlated with more aggressive disease course, higher risk of death, and shorter median overall survival (OS) (48).

On a histopathologic level, several classification schemes have been utilized to predict prognosis and thus guide clinical management, including Folpe (33), Bleeker (34), modified Folpe, Bennet, Schoolmaster (49), and WHO (30), but entail variability in delineating benign/non-malignant versus malignant PEComas (4). A systematic review of uterine PEComas by Garzon et al. identified the modified Folpe classification as the most accurate in predicting the behavior of PEComas, but they proposed changing the requisite cutoffs for tumor size from ≥5 to ≥8 cm and mitoses from ≥1/50 hpf to ≥5/50 hpf to improve accuracy (49). Based on retrospective clinicopathologic analysis, Gantzer et al. successfully developed and validated a PEComa prognostic score (PEC-PRO) to reliably predict event-free survival after surgical resection; the PEC-PRO score also directly correlated with the Folpe classification (50).

Although PEComas are typically diagnosed incidentally, they can present with pain or discomfort associated with the tumor and weight loss (38), similar to our patient’s presentation. Radiographic findings of PEComas are variable, but CT can show intense post-contrast enhancement due to abundant vascular stroma, a finding present in our patient’s initial CT neck and chest (51). Contrast-enhanced Doppler ultrasonography can show a heterogeneous, hypoechoic lesion with peripheral vascularization (21, 23). On MRI, common features include well-circumscribed tumors with no infiltration or local invasion, calcification and/or hemorrhage, hypo- to isointensity relative to skeletal muscle on T1-weighted imaging, heterogeneous hyperintensity on T2-weighted imaging, and significant post-gadolinium enhancement (12, 52). While PEComas, especially those of gastrointestinal origin, tend to exhibit intratumoral hemorrhage on imaging due to their vascularity (53), our patient developed severe anemia without such signs of clinical blood loss, a phenomenon described by other case reports (12). It is likely that his acute on chronic anemia was secondary to exacerbation of anemia of chronic disease due to malignant disease progression, with contribution to anemia from sirolimus therapy (54). Interestingly, PEComas can display FDG avidity on PET/CT, which may not be interpreted as malignant due to their typically benign nature, masking a subset of PEComas with malignant potential (12, 51, 55). Given the heterogeneity in imaging findings, there is no consensus on posttreatment imaging surveillance. However, patients at greater risk have been surveilled with serial PET/CT over the years (56).

Malignant PEComas are primarily treated with radical surgical resection due to resistance to chemotherapy and RT (12, 20, 36, 38). Due to the rarity of malignant PEComas, there are limited data on surgical technique, but two factors have been identified as negative prognosticators by a retrospective cohort study: 1) an intraoperative period between primary tumor and first pulmonary metastasis of less than 30 months and 2) high histologic grade (57). Additional negative prognosticators include the presence of metastasis on diagnosis, grouped-Bleeker’s risk category, and, within the metastatic patient population, the presence of lymph node metastasis (4) due to the importance of surgical resection in treatment. In several cases, metastasectomy of metastatic foci (i.e., lungs, liver, retroperitoneum) permitted durable long-term disease control, with Dudek et al. showing a 5-year OS of 40.4% after the first pulmonary metastasis (57). As such, surgery should always be considered for countable and resectable metastases, similar to other sarcomas (36, 58). However, one case report described a good response to neoadjuvant chemoradiation for an upper extremity PEComa with six cycles of doxorubicin plus ifosfamide followed by preoperative RT to 50 Gy, causing 20% tumor necrosis prior to limb-sparing wide local excision (59). Additionally, neoadjuvant epirubicin with cisplatin and ifosfamide decreased tumor size and improved resectability in a patient with a uterine PEComa (60). Nonetheless, the benefit of neoadjuvant chemotherapy with ifosfamide, vincristine, and dactinomycin has been described as limited to devascularizing the tumor without decreasing tumor size (61); other cytotoxic chemotherapeutics exhibit a small objective response (38). While the role of RT in treating malignant PEComas appears limited due to the paucity of literature, preoperative ultrahypofractionated stereotactic body RT (SBRT) to 60 Gy in 8 fractions using 4D CT and MRI planning permitted a margin-negative resection of a liver PEComa initially invading the inferior vena cava with local control close to 2 years (62). In the adjuvant setting, surgical resection of an adrenal PEComa with unclear margin status followed by conventionally fractionated IMRT to 46.8 Gy in 26 fractions to the tumor bed also resulted in local control close to 2 years (63). In another case, two courses of SBRT up to 30 Gy in 6 fractions coupled with the programmed death (PD)-1 inhibitor tislelizumab provided disease control for a metastatic pelvic PEComa with positive PD-L1 expression (64); utilization of the SBRT technique in conjunction with SIBs appears to permit dose escalation to overcome the inherent radioresistance of malignant PEComas while sparing normal tissues (65). In a case described by McBride et al., pembrolizumab provided a complete response for recurrent PD-L1-positive PEComa metastatic to the lungs status post-lobectomy (66). Thus, the coordination of dose-escalated RT, surgery in operable patients, and immunotherapy may represent the clinical amalgam needed to improve both local and distant control as there appears to be limited overall benefit with cytotoxic chemotherapies, but such determinations require prospective randomized controlled trials.

Due to aberrant mTOR signaling, mTOR inhibitors have demonstrated efficacy in treating malignant PEComas with the phase II AMPECT trial showing median OS of 40.8 months and progression-free survival (PFS) of 10.6 months (67). Consequently, the National Comprehensive Cancer Network (NCCN) guidelines recommend albumin-bound sirolimus for unresectable locally advanced disease or metastatic disease; the intravenous administration of nanoparticle protein-bound sirolimus showed higher intratumoral accumulation, mTOR inhibition, and tumor growth inhibition compared to oral mTOR inhibitors (67, 68). A subsequent retrospective cohort study showed a 5-year OS of 65% over 55.7 months with albumin-bound sirolimus in patients with metastatic PEComa ineligible for surgery (69). However, sirolimus, everolimus, and temsirolimus remain the NCCN guideline-recommended systemic therapies, with sirolimus employed in the present case. Hypofractionated palliative RT to 24 Gy in 8 fractions delivered with concurrent sirolimus provided excellent intrathoracic disease control in a patient with metastatic PEComa thought to be of pulmonary primary, although there was a mixed response to sirolimus at distant metastatic sites (70). Everolimus was able to stabilize disease in a patient with recurrent metastatic PEComa (71). Temsirolimus utilized as adjuvant therapy after lobectomy for metastatic PEComa resulted in durable disease-free survival in a patient with a malignant uterine PEComa initially treated with surgical resection (72). Unfortunately, mTOR inhibitors do not appear to improve PFS relative to standard chemotherapy regimens or provide OS benefit in *TSC1/TSC2* mutated malignant PEComas (38, 73). Thus, refractory cases have been treated with the VEGF inhibitors pazopanib and apatinib as salvage or combination therapy (46, 47, 74, 75) but with overall poor objective response and PFS typically less than 6 months.

## Conclusions

In the present case, the patient developed mediastinal invasion and replacement of the right upper and middle lungs despite oral sirolimus and definitive hypofractionated RT, an aggressive presentation not previously described in the literature to our knowledge. While cases of progression on mTOR inhibitors have been documented, this case highlights how aggressive malignant PEComas invading the mediastinum can behave and the need for more population-based studies to guide treatment paradigms in inoperable patients. Although successful in other case reports, ultrahypofractionated SBRT would have been difficult to employ over such large treatment volumes in the mediastinum due to the proximity of dose-limiting structures such as the lungs, esophagus, and spinal cord. As our patient’s biopsy specimen was not tested for PD-L1 expression, it is unclear if immunotherapy would have represented a viable alternative salvage treatment, but, nonetheless, salvage treatment with pazopanib could not be implemented to determine the patient’s relative response to VEGF inhibitors.

## Data Availability

The original contributions presented in the study are included in the article/Supplementary Material. Further inquiries can be directed to the corresponding author.

## Glossary

PEComa: perivascular epithelioid cell tumor
PEComa-NOS: Perivascular epithelioid cell tumor-not otherwise specified
RT: radiotherapy
WHO: World Health Organization
TS: tuberous sclerosis
HPF: High-power field
IHC: immunohistochemistry
HMB-45: human melanoma black 45
Melan-A: melanoma antigen
CT: computed tomography
PET: positron emission tomography
FDG: ^18^F-2-deoxy-D-glucose
IMRT: intensity-modulated radiotherapy
Gy: Gray
SIB: simultaneous integrated boost
EQD2: equivalent dose in 2 Gy fractions
MRI: magnetic resonance imaging
PEG: percutaneous endoscopic gastrostomy
mTOR: mammalian target of rapamycin
VEGF: vascular endothelial growth factor
OS: overall survival
SBRT: stereotactic body radiotherapy
PD-L1: programmed death ligand
PFS: progression-free survival
NCCN: National Comprehensive Cancer Network
